# Synthesis and Bioactivities Study of Novel Pyridylpyrazol Amide Derivatives Containing Pyrimidine Motifs

**DOI:** 10.3389/fchem.2020.00522

**Published:** 2020-07-31

**Authors:** Wenneng Wu, Meihang Chen, Qiang Fei, Yonghui Ge, Yingying Zhu, Haijiang Chen, Maofa Yang, Guiping Ouyang

**Affiliations:** ^1^Food and Pharmaceutical Engineering Institute, Guiyang University, Guiyang, China; ^2^Center for Research and Development of Fine Chemicals, School of Pharmaceutical Sciences, Entomology of Institute, Guizhou University, Guiyang, China; ^3^Material and Chemistry Engineering Institute, Tongren College, Tongren, China; ^4^School of Chemical Engineering, Guizhou Institute of Technology, Guiyang, China

**Keywords:** pyridylpyrazol, amide, pyrimidine, antifungal activity, insecticidal activity

## Abstract

In this study, thirteen new pyridylpyrazolamide derivatives containing pyrimidine motifs were synthesized via six-step reactions. Bioassay results showed that some of the synthesized compounds revealed good antifungal properties against *Sclerotinia sclerotiorum, Phytophthora infestans, Thanatephorus cucumeris, Gibberella zeae, Fusarium oxysporum, Cytospora mandshurica, Botryosphaeria dothidea*, and *Phompsis* sp. at 50 μg/mL, which were similar to those of Kresoxim-methyl or Pyrimethanil. Meanwhile, bioassay results indicated that the synthesized compounds showed a certain insecticidal activity against *Spodoptera litura, Mythimna separata, Pyrausta nubilalis, Tetranychus urticae, Rhopalosiphum maidis*, and *Nilaparvata lugens* at 200 μg/mL, which was lower than that of Chlorantraniliprole. To the best of our knowledge, this study is the first report on the antifungal and insecticidal activities of pyridylpyrazol amide derivatives containing a pyrimidine moiety.

## Introduction

Plant fungal and insect diseases have posed serious threats to crops in the world and caused a severe loss throughout the world (Strange and Scott, 2005; Yang et al., 2015). Nowadays, some of the available traditional fungicides and insecticides, such as Kresoxim-methyl, Pyrimethanil, Chlorantraniliprole, etc., are widely used to prevent plant harmful fungal and insect diseases. However, prolonged use of traditional pesticides can not only lead to drug resistance, but also have a harmful influence on the safety of the plants and the environment. Therefore, the development of novel and promising fungicides and insecticides is still an urgent task.

**Figure 1:**
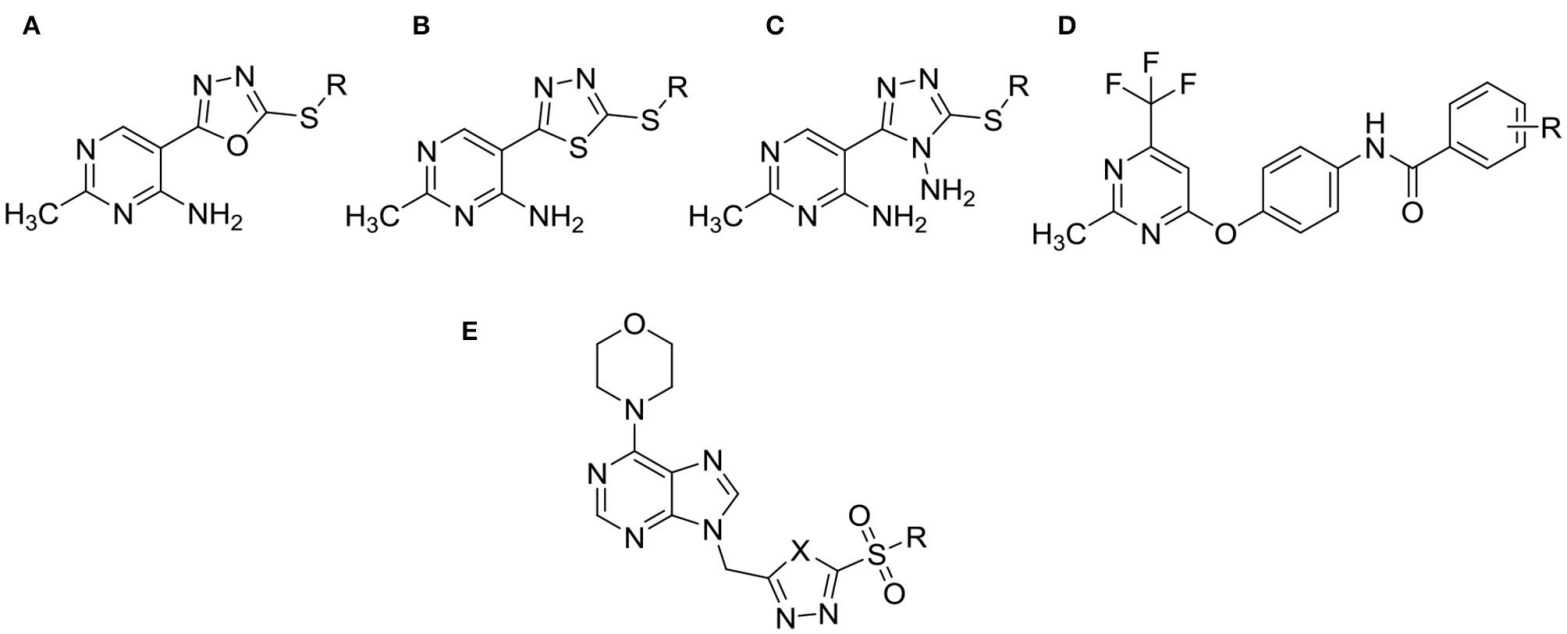
The structures of the pyrimidine derivatives containing 1,3,4-oxadiazole **(A,E)**, 1,3,4-thiadiazole **(B,E)**, 1,2,4-triazole **(C)** or amide **(D)** moiety reported by our research group.

**Figure 2:**
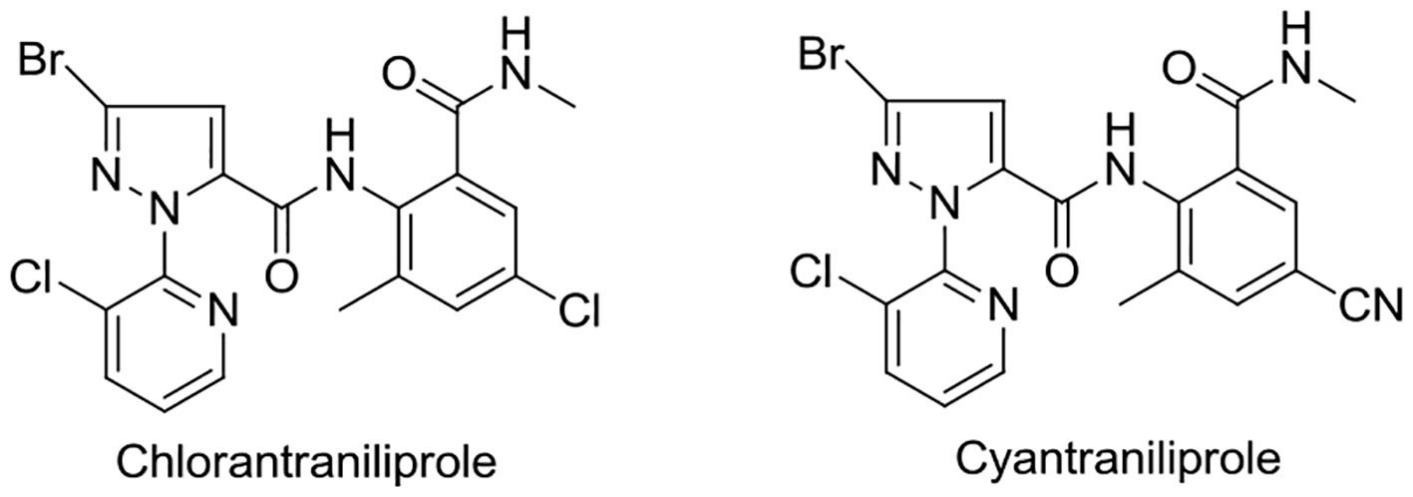
Structures of chlorantraniliprole and cyantraniliprole.

**Figure 3:**
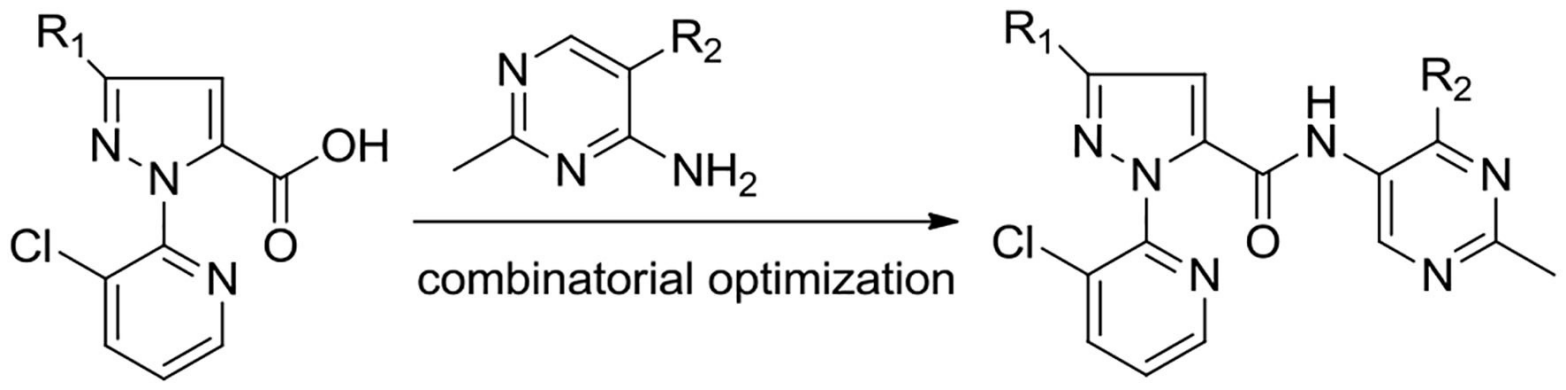
Design strategy for title compounds **6a**–**6m**.

Pyrimidine derivatives, which play an important role in synthesis of various active molecules, have versatile properties in modern life, such as antifungal (Chen et al., 2008; Zhang et al., 2016), antibacterial (Triloknadh et al., 2018; Fang et al., 2019), insecticidal (Liu et al., 2017; Shen et al., 2018), herbicidal (Chen et al., 2015; Li et al., 2018), and antiviral (Xu et al., 2015; Wang Y. Y. et al., 2018) activities. In the previous work, some of the pyrimidine derivatives (for example, Mepanipyrim, Pyrimethanil, Diflumetorim, Azoxystrobin, and so on), which were known for their abilities to control severe fungal diseases, have been marketed as commercial pesticides worldwide. Meanwhile, in our preliminary work, several series of pyrimidine derivatives containing 1,3,4-oxadiazole (Figures 1A,E), 1,3,4-thiadiazole (Figures 1B,E), 1,2,4-triazole (Figure 1C) or amide (Figure 1D) moiety, as shown in Figure 1, were reported and revealed better antiviral, antifungal, and antibacterial activities (Wu et al., 2015, 2016a,b, 2019a,b).

In recent years, pyridylpyrazole amide derivatives have attracted more and more considerable attention owing to their broad class of biological activities in pesticide chemistry, such as antifungal (Yan et al., 2012; Wang B. L. et al., 2018) and insecticidal (Wang et al., 2013, 2019; Shi et al., 2017; Wang B. L. et al., 2018) activity. In the past few years, some representative examples of pyridylpyrazole amide derivatives (for example, chlorantraniliprole and cyantraniliprole) were commercialized as pesticides, as shown in Figure 2.

To develop effective pesticide agents, we aim to introduce the pyrimidine ring to the pyridiylpyrazol amide skeleton to design a series of novel pyridiylpyrazol amide derivatives containing a pyrimidine moiety (Figure 3). As far as we know, it is the first report on the antifungal and insecticidal activities of pyridylpyrazol amide derivatives containing a pyrimidine moiety.

## Experimental and Methods

### General Information

JEOL-ECX 500 NMR spectrometer (JEOL, Tokyo, Japan) was used to analyse the NMR spectral (^1^H NMR and ^13^C NMR) at room temperature using TMS as an internal standard and DMSO-*d*_6_ as the solvent. Elemental analysis was performed on the Elementar Vario-III CHN analyser (Elementar, Hanau, Germany). Mass spectral were conducted on the Agilent 5973 organic mass spectrometer (Agilent Technologies, Palo Alto, CA, USA). Melting points were determined on the XT-4 binocular microscope (Beijing Tech Instrument Co., China). All commercial reagents and solvents were used as they did not require any purification before use.

### Synthesis

#### Preparation Procedure of the Key Intermediate 5

**Scheme 1:**
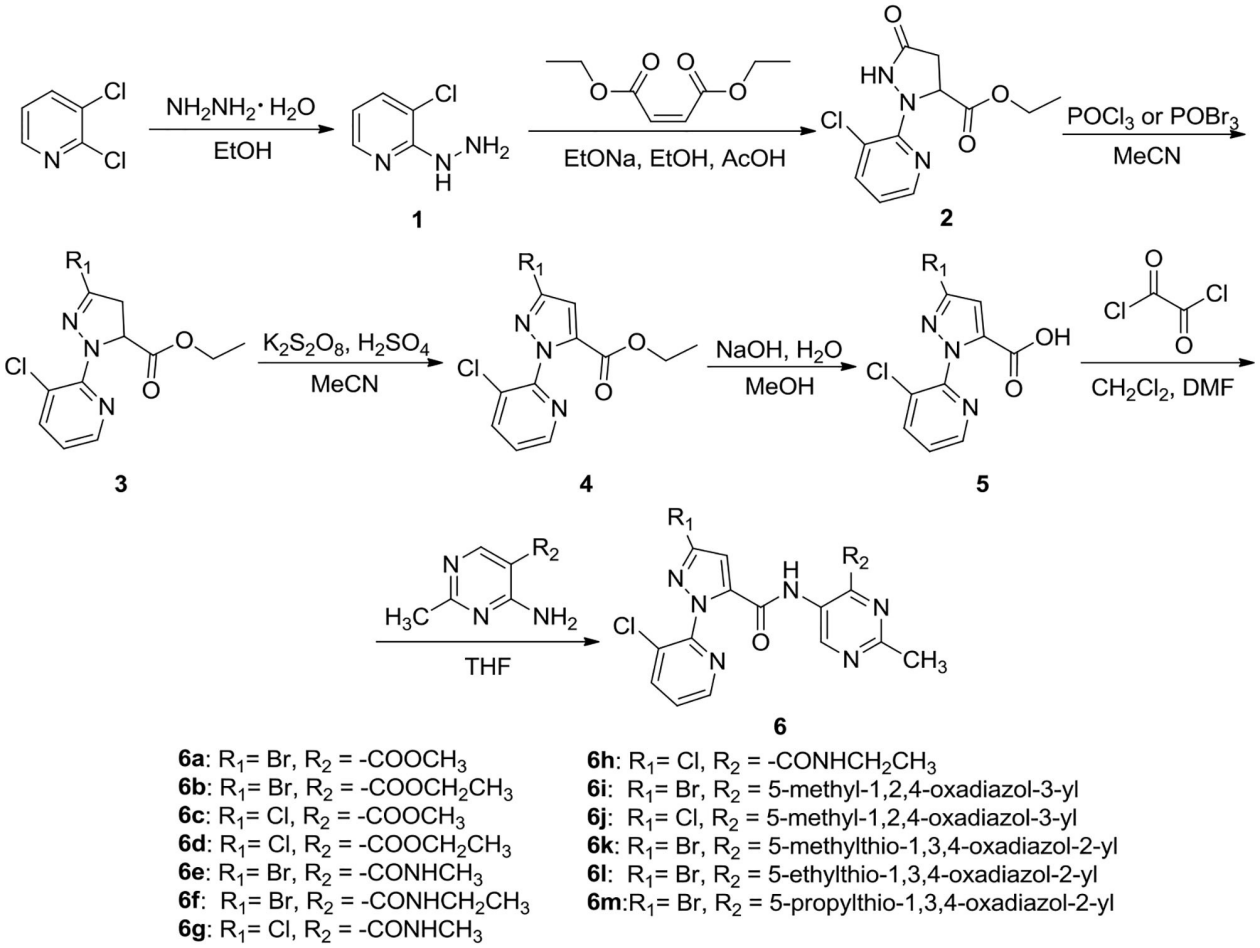
Synthetic route of the title compounds **6a**–**6m**.

The synthetic procedure for the key intermediate **5** is shown in Scheme 1. To a mixture of 2,3-dichloropyridine (0.1 mol) dissolved in anhydrous ethanol (120 mL), 80% hydrazine hydrate (80 mL) was added dropwise and reacted under reflux. Upon completion of the reaction, the reaction solution was cooled to room temperature and the solvent was removed under reduced pressure. The residue was washed with water and recrystallized with ethanol to gain intermediate **1**. Intermediate **1** (90 mmol) and diethyl maleate (90 mmol) were added to the mixture of sodium ethoxide (90 mmol) and ethanol (100 mL), then a moderate amount of glacial acetic acid was added when the temperature was below 60°C. Upon completion of the reaction, the reaction solution was poured into 100 mL distilled water to precipitate the solid, then the solid was recrystallized with ethanol to obtain intermediate **2**. Then, a mixture of intermediate **2** (45 mmol), phosphorus oxychloride (POCl_3_, 50 mmol) or phosphorus oxybromide (POBr_3_, 50 mmol), and acetonitrile (CH_3_CN, 50 mL) was reacted under reflux. After ending the reaction, the reaction mixture was poured into 30 mL distilled water, extracted with dichloromethane (CH_2_Cl_2_), and dried with anhydrous sodium sulfate (Na_2_SO_4_) to give intermediate **3** (Wang B. L. et al., 2018). After that, a mixture of intermediate **3** (40 mmol), potassium persulfate (K_2_S_2_O_8_, 44 mmol), and CH_3_CN (50 mL) reacted under reflux. Upon completion of the reaction, the reaction mixture was poured into 100 mL of distilled water. The residue was washed with water and recrystallized with ethanol to obtain intermediate **4** (Wang B. L. et al., 2018). Finally, sodium hydroxide (NaOH, 30 mmol) dissolved in 10 mL of water was added to the mixture of intermediate **4** and methanol (20 mL) and reacted under reflux. When the reaction was completed, the reaction mixture was poured into 100 mL of distilled water and acidified the mixture to pH 5–6 using concentrated hydrochloric acid. The key intermediate **5** was attained after recrystallization with ethanol. ^1^H NMR spectral data for intermediates **1–5** are reported in the Supplementary Data.

#### General Procedure for the Preparation of the Target Compounds 6a−6m

As shown in Scheme 1, oxalyl chloride (2 mL) and 4 drops of *N, N*-dimethylformamide (DMF) were added to a mixture of intermediate **5** dissolved in 10 mL CH_2_Cl_2_, and reacted at room temperature. When the reaction was completed, the CH_2_Cl_2_ and excess oxaloyl chloride were removed under reduced pressure. Then, 5-substituted-4-amino-2-methylpyrimidine was added to the residue dissolved in 5 mL tetrahydrofuran (THF) and reacted under reflux. At the end of the reaction, the reaction mixture was poured into 20 mL of water, extracted with ethyl acetate, and recrystallized with ethanol to obtain the title compounds **6a**–**6m**.

Methyl4-(3-bromo-1-(3-chloropyridin-2-yl)-1*H*-pyrazole-5-carboxamido)-2-methyl-pyrimidine-5-carboxylate (**6a**). White solid; yield 45.1%; m.p. 220–222°C; ^1^H NMR (DMSO-*d*_6_, 500 MHz) δ: 11.85 (s, 1H, CONH), 8.84 (s, 1H, Pyrimidine-H), 8.52 (d, 1H, *J* = 4.6 Hz, Pyridine-H), 8.23 (d, 1H, *J* = 6.9 Hz, pyridine-H), 7.67 (dd, 1H, *J*_1_ = 4.55 Hz, *J*_2_ = 8.0 Hz,pyridine-H), 7.58 (s, 1H, Pyrazole-H), 3.61 (s, 3H), 2.62 (s, 3H);^13^C NMR (DMSO-*d*_6_, 125 MHz) δ: 170.51, 165.33, 159.38, 156.62, 154.98, 148.73, 147.82, 140.40, 140.06, 138.27,1 128.50, 127.55, 115.07, 109.32, 52.83, 26.00; MS (ESI) m/z: 451.1 ([M+H]^+^); Anal. Calcd. for C_16_H_12_BrClN_6_O_3_: C 42.55, H 2.68, N 18.61; found: C 42.60, H 2.70, N 18.66.

Ethyl4-(3-bromo-1-(3-chloropyridin-2-yl)-1*H*-pyrazole-5-carboxamido)-2-methyl-pyrimidine-5-carboxylate (**6b**). White crystals; yield 50.4%; m.p. 176–177°C; ^1^H NMR (DMSO-*d*_6_, 500 MHz) δ: 11.88 (s, 1H, CONH), 8.84 (s, 1H, Pyrimidine-H), 8.51 (d, 1H, *J* = 4.0 Hz, Pyridine-H), 8.24 (d, 1H, *J* = 8.0 Hz, pyridine-H), 7.66 (dd, 1H, *J*_1_ = 4.55 Hz, *J*_2_ = 8.0 Hz, pyridine-H), 7.59 (s, 1H, Pyrazole-H), 4.09 (q, 2H, *J* = 6.9 Hz), 2.64 (s, 3H), 1.08 (t, 3H, *J* = 7.45 Hz); ^13^C NMR (DMSO-*d*_6_, 125 MHz) δ: 170.51, 164.80, 159.38, 156.64, 148.73, 147.80, 140.33, 140.07, 138.27, 128.46, 127.52, 115.38, 109.34, 61.68, 25.97, 14.32; MS (ESI) m/z: 465.1 ([M+H]^+^); Anal. Calcd. for C_17_H_14_BrClN_6_O_3_: C 43.85, H 3.03, N 18.05; found: C 43.90, H 3.00, N 18.06.

Methyl4-(3-chloro-1-(3-chloropyridin-2-yl)-1*H*-pyrazole-5-carboxamido)-2-methyl-pyrimidine-5-carboxylate (**6c**). White solid; yield 48.2%; m.p. 213–215°C; ^1^H NMR (DMSO-*d*_6_, 500 MHz) δ: 11.87 (s, 1H, CONH), 8.85 (s, 1H, Pyrimidine-H), 8.53 (d, 1H, *J* = 2.85 Hz, Pyridine-H), 8.25 (d, 1H, *J* = 7.65 Hz, pyridine-H), 7.68 (dd, 1H, *J*_1_ = 2.85 Hz, *J*_2_ = 8.0 Hz, pyridine-H), 7.55 (s, 1H, Pyrazole-H), 3.62 (s, 3H), 2.64 (s, 3H); ^13^C NMR (DMSO-*d*_6_, 125 MHz) δ: 170.54, 165.32, 159.41, 156.62, 155.01, 148.71, 147.85, 140.48, 140.01, 138.30, 128.51, 127.54, 115.05, 109.30, 52.82, 26.01; MS (ESI) m/z: 407.1 ([M+H]^+^); Anal. Calcd. for C_16_H_12_Cl_2_N_6_O_3_: C 47.19, H 2.97, N 20.64; found: C 47.20, H 3.00, N 20.62.

Ethyl4-(3-chloro-1-(3-chloropyridin-2-yl)-1*H*-pyrazole-5-carboxamido)-2-methyl-pyrimidine-5-carboxylate (**6d**). Yellow crystals; yield 38.5%; m.p. 191–192°C; ^1^H NMR (DMSO-*d*_6_, 500 MHz) δ: 11.88 (s, 1H, CONH), 8.84 (s, 1H, Pyrimidine-H), 8.51 (d, 1H, *J* = 2.85Hz, Pyridine-H), 8.23 (d, 1H, *J* = 7.65 Hz, pyridine-H), 7.66 (dd, 1H, *J*_1_ = 2.85 Hz, *J*_2_ = 8.00 Hz, pyridine-H), 7.53 (s, 1H, Pyrazole-H), 4.06 (q, 2H, *J* = 6.90 Hz), 2.64 (s, 3H), 1.08 (t, 3H, *J* = 6.85 Hz); ^13^C NMR (DMSO-*d*_6_, 125 MHz) δ: 170.52, 164.80, 159.38, 156.64, 148.73, 147.80, 140.33, 140.07, 138.27, 128.46, 127.52, 115.38, 109.34,61.68, 25.97,14.32; MS (ESI) m/z: 422.1 ([M+H]^+^); Anal. Calcd. for C_17_H_14_Cl_2_N_6_O_3_: C 48.47, H 3.35, N 19.95; found: C 48.50, H 3.40, N 20.02.

4-(3-Bromo-1-(3-chloropyridin-2-yl)-1*H*-pyrazole-5-carboxamido)-*N*-2-dimethylpyrimidine-5-carboxamide (**6e**). White solid; yield 52.6%; m.p. 228–233°C; ^1^H NMR (DMSO-*d*_6_, 500 MHz) δ: 12.33 (s, 1H, CONH), 8.78 (s, 1H, Pyrimidine-H), 8.74 (d, 1H, *J* = 4.55 Hz, CONH), 8.48 (d, 1H, *J* = 4.00 Hz, Pyridine-H), 8.19 (d, 1H, *J* = 8.05Hz, Pyridine-H), 7.62 (dd, 1H, *J*_1_ = 4.60 Hz, *J*_2_ = 8.00 Hz, Pyridine-H), 7.32 (s, 1H, Pyrazole-H), 2.67 (d, 3H, *J* = 4.6 Hz), 2.45 (s, 3H); ^13^C NMR (DMSO-*d*_6_, 125 MHz)δ: 169.38, 165.98, 157.28, 156.02, 154.80, 148.58, 147.82, 140.09, 139.38, 128.40, 127.68, 127.48, 114.27, 111.72, 26.67, 26.07; MS (ESI) m/z: 450.1 ([M+H]^+^); Anal. Calcd. for C_16_H_13_BrClN_7_O_2_: C 42.64, H 2.91, N 21.76; found: C 42.66, H 2.90, N 21.77.

4-(3-Bromo-1-(3-chloropyridin-2-yl)-1*H*-pyrazole-5-carboxamido)-*N*-ethyl-2-methylpyrimidine-5-carboxamide (**6f**). White solid; yield 60.1%; m.p. 236–237°C; ^1^H NMR (DMSO-*d*_6_, 500 MHz) δ: 12.33 (s, 1H, CONH), 8.78 (s, 1H, Pyrimidine-H), 8.72 (s, 1H, CONH), 8.48 (d, 1H, *J* = 4.00 Hz, Pyridine-H), 8.19 (d, 1H, *J* = 8.05 Hz, Pyridine-H), 7.62 (dd, 1H, *J*_1_ = 4.60 Hz, *J*_2_ = 8.00 Hz, Pyridine-H), 7.32 (s, 1H, Pyrazole-H), 2.84 (q, 2H, *J* = 6.85 Hz), 2.45 (s, 3H), 1.04 (t, 3H, *J* = 6.25 Hz); ^13^C NMR (DMSO-*d*_6_, 125 MHz)δ: 169.35, 166.00, 157.31, 156.04, 154.78, 148.60, 147.81, 140.11, 139.35, 128.42, 127.65, 127.49, 114.27, 111.76, 26.66, 26.03, 15.32; MS (ESI) m/z: 464.1 ([M+H]^+^); Anal. Calcd. for C_17_H_15_BrClN_7_O_2_: C 42.64, H 2.91, N 21.76; found: C 42.66, H 2.90, N 21.77.

4-(3-Chloro-1-(3-chloropyridin-2-yl)-1*H*-pyrazole-5-carboxamido)-*N*,2-dimethylpyrimidine-5-carboxamide (**6g**). White solid; yield 45.9%; m.p. 213–215°C; ^1^H NMR (DMSO-*d*_6_, 500 MHz) δ: 11.87 (s, 1H, CONH), 8.85 (s, 1H, Pyrimidine-H), 8.71 (s, 1H, CONH), 8.53 (d, 1H, *J*= 2.85 Hz, Pyridine-H), 8.25 (d, 1H, *J* = 7.65 Hz, Pyridine-H), 7.68 (dd, 1H, *J*_1_ = 2.85 Hz, *J*_2_ = 8.0 Hz, Pyridine-H), 7.55 (s, 1H, Pyrazole-H), 2.85 (s, 3H), 2.64 (s, 3H); ^13^C NMR (DMSO-*d*_6_, 125 MHz) δ: 170.51, 165.33, 159.38, 156.62, 154.98, 148.73, 147.82, 140.40, 140.06, 138.27, 128.50, 127.55, 115.07, 109.32, 52.83, 26.62, 26.04; MS (ESI) m/z: 406.1 ([M+H]^+^); Anal. Calcd. for C_16_H_13_Cl_2_N_7_O_2_: C 47.31, H 3.23, N 24.14; found: C 47.32, H 3.20, N 24.12.

4-(3-Chloro-1-(3-chloropyridin-2-yl)-1*H*-pyrazole-5-carboxamido)-*N*-ethyl-2-methylpyrimidine-5-carboxamide (**6h**). Yellow crystals; yield 47.8%; m.p. 191–192°C; ^1^H NMR (DMSO-*d*_6_, 500 MHz) δ: 11.88 (s, 1H, CONH), 8.84 (s, 1H, Pyrimidine-H), 8.71 (s, 1H, CONH), 8.51 (d, 1H, *J* = 2.85 Hz, Pyridine-H), 8.23 (d, 1H, *J* = 7.65 Hz, pyridine-H), 7.66 (dd, 1H, *J*_1_ = 2.85 Hz, *J*_2_ = 8.00 Hz, pyridine-H), 7.53 (s, 1H, Pyrazole-H), 4.06 (q, 2H, *J* = 6.90 Hz), 2.64 (s, 3H), 1.08 (t, 3H, *J* = 6.85 Hz); ^13^C NMR (DMSO-*d*_6_, 125 MHz) δ: 170.52, 164.89, 159.38, 156.64, 148.73, 147.80, 140.33, 140.07, 138.27, 128.46, 127.52, 115.38, 109.34, 61.68, 25.97,15.32; MS (ESI) m/z: 420.1 ([M+H]^+^); Anal. Calcd. for C_17_H_15_Cl_2_N_7_O_2_: C 48.59, H 3.60, N 23.33; found: C 48.61, H 3.62, N 23.30.

3-Bromo-1-(3-chloropyridin-2-yl)-*N*-(2-methyl-5-(5-methyl-1,2,4-oxadiazol-3-yl)pyrimidin-4-yl)-1*H*-pyrazole-5-carboxamide (**6i**). Yellow crystals; yield 36.5%; m.p. 180–181°C; ^1^H NMR (DMSO-*d*_6_, 500 MHz) δ: 11.64 (s, 1H, CONH), 8.99 (s, 1H, Pyrimidine-H), 8.49 (d, 1H, *J* = 4.55 Hz, Pyridine-H), 8.18 (d, 1H, *J* = 8.05 Hz, Pyridine-H), 7.63 (dd, 1H, *J*_1_ = 4.60 Hz, *J*_2_ = 8.0 Hz, Pyridine-H), 7.54 (s, 1H, Pyrazole-H), 2.67 (s, 3H), 2.61 (s, 3H); ^13^C NMR (DMSO-*d*_6_, 125 MHz)δ: 177.49, 169.89, 164.89, 158.92, 155.80, 155.08, 148.68, 147.69, 139.94, 138.68, 128.48, 127.52, 127.41, 112.34, 111.50, 25.96, 12.43; MS (ESI) m/z: 475.1 ([M+H]^+^); Anal. Calcd. for C_17_H_12_BrClN_8_O_2_: C 42.92, H 2.54, N 23.56; found: C 42.91, H 2.50, N 23.60.

3-Chloro-1-(3-chloropyridin-2-yl)-*N*-(2-methyl-5-(5-methyl-1,2,4-oxadiazol-3-yl)pyrimidin-4-yl)-1*H*-pyrazole-5-carboxamide (**6j**). Yellow crystals; yield 41.3%; m.p. 151–152°C; ^1^H NMR (DMSO-*d*_6_, 500 MHz) δ: 11.64 (s, 1H, CONH), 8.99 (s, 1H, Pyrimidine-H), 8.49 (d, 1H, *J* = 4.55 Hz, Pyridine-H), 8.18 (d, 1H, *J* = 8.05 Hz, Pyridine-H), 7.63 (dd, 1H, *J*_1_ = 4.60 Hz, *J*_2_ = 8.0 Hz, Pyridine-H), 7.54 (s, 1H, Pyrazole-H), 2.67 (s, 3H), 2.61 (s, 3H); ^13^C NMR (DMSO-*d*_6_, 125 MHz)δ: 177.52, 169.87, 164.92, 158.94, 155.85, 155.11, 148.65, 147.72, 139.99, 138.71, 128.50, 127.53, 127.45, 112.36, 111.32, 26.02, 12.45; MS (ESI) m/z: 431.1 ([M+H]^+^); Anal. Calcd. for C_17_H_12_BrClN_8_O_2_: C 47.35, H 2.80, N 25.98; found: 47.37, H 2.77, N 25.99.

3-Bromo-1-(3-chloropyridin-2-yl)-*N*-(2-methyl-5-(5-(methylthio)-1,3,4-oxadiazol-2-yl)pyrimidin-4-yl)-1*H*-pyrazole-5-carboxamide (**6k**). Yellow crystals; yield 46.8%; m.p. 166–168°C; ^1^H NMR (DMSO-*d*_6_, 500 MHz) δ: 11.84 (s, 1H, CONH), 9.04 (s, 1H, Pyrimidine-H), 8.46 (d, 1H, *J* = 5.15 Hz, Pyridine-H), 8.18 (d, 1H, *J* = 8.0 Hz, Pyridine-H), 7.62 (dd, 1H, *J*_1_ = 4.55 Hz, *J*_2_ = 7.7 Hz, Pyridine-H), 7.58 (s, 1H, Pyrazole-H), 2.72 (s, 3H), 2.66 (s, 3H); ^13^C NMR (DMSO-*d*_6_, 125 MHz) δ: 170.30, 164.72, 162.00, 158.79, 156.05, 148.51, 148.10, 147.81, 140.01, 138.54, 128.35, 127.60, 127.42, 112.61, 108.46, 26.10, 15.62; MS (ESI) m/z: 507.1 ([M+H]^+^); Anal. Calcd. for C_17_H_12_BrClN_8_O_2_S: C 40.21, H 2.38, N22.07; found: C 40.24, H 2.40, N22.05.

3-Bromo-1-(3-chloropyridin-2-yl)-*N*-(5-(5-(ethylthio)-1,3,4-oxadiazol-2-yl)-2-methylpyrimidin-4-yl)-1*H*-pyrazole-5-carboxamide (**6l**). Yellow crystals; yield 42.5%; m.p. 176–177°C; ^1^H NMR (DMSO-*d*_6_, 500 MHz) δ: 11.84 (s, 1H, CONH), 9.04 (s, 1H, Pyrimidine-H), 8.46 (d, 1H, *J* = 5.15 Hz, Pyridine-H), 8.18 (d, 1H, *J* = 8.0 Hz, Pyridine-H), 7.62 (dd, 1H, *J*_1_ = 4.55 Hz, *J*_2_ = 7.7 Hz, Pyridine-H), 7.58 (s, 1H, Pyrazole-H), 3.12 (q, 2H, *J* = 6.9 Hz), 2.66 (s, 3H), 1.35 (t, 3H, *J* = 7.45 Hz); ^13^C NMR (DMSO-*d*_6_, 125 MHz) δ: 170.25, 164.74, 161.95, 158.75, 156.08, 148.25, 148.12, 147.69, 140.07, 138.55, 128.27, 127.60, 127.44, 112.63, 108.56, 27.86, 26.10, 14.23; MS (ESI) m/z: 521.1 ([M+H]^+^); Anal. Calcd. for C_18_H_14_BrClN_8_O_2_S: C 41.43, H 2.70, N 21.48; found: C 41.45, H 2.74, N 21.49.

3-Bromo-1-(3-chloropyridin-2-yl)-N-(2-methyl-5-(5-(propylthio)-1,3,4-oxadiazol-2-yl)pyrimidin-4-yl)-1*H*-pyrazole-5-carboxamide (**6m**). Yellow crystals; yield 39.8%; m.p. 197–199°C; ^1^H NMR (DMSO-*d*_6_, 500 MHz) δ: 11.84 (s, 1H, CONH), 9.04 (s, 1H, Pyrimidine-H), 8.46 (d, 1H, *J*=5.15 Hz, Pyridine-H), 8.18 (d, 1H, *J* = 8.0 Hz, Pyridine-H), 7.62 (dd, 1H, *J*_1_ = 4.55 Hz, *J*_2_ = 7.7 Hz, Pyridine-H), 7.58 (s, 1H, Pyrazole-H), 3.15 (t, 2H, *J* = 6.9 Hz), 2.66 (s, 3H), 1.68 (m, 2H), 0.95 (t, 3H, *J* = 7.45 Hz); ^13^C NMR (DMSO-*d*_6_, 125 MHz)δ: 170.31, 164.70, 162.03, 158.75, 156.02, 148.48, 148.07, 147.77, 140.04, 138.50, 128.32, 127.60, 127.44, 112.67, 108.49, 34.43, 26.10, 22.92, 13.33; MS (ESI) m/z: 535.1 ([M+H]^+^); Anal. Calcd. for C_19_H_16_BrClN_8_O_2_S: C 42.59, H 3.01, N 20.91; found: C 42.53, H 3.00, N 20.92.

#### Antifungal Biological Assay

The antifungal activities of the title compounds **6a**–**6m** against *Sclerotinia sclerotiorum* (*S. sclerotiorum*), *Phytophthora infestans* (*P. infestans*), *Thanatephorus cucumeris* (*T. cucumeris*), *Gibberella zeae* (*G. zeae*), *Fusarium oxysporum* (*F. oxysporum*), *Cytospora mandshurica* (*C. mandshurica*), *Botryosphaeria dothidea* (*B. dothidea*), and *Phompsis* sp. were evaluated at the concentration of 50 μg/mL (Min et al., 2016; Wu et al., 2019a,b). The target compounds **6a**–**6m** (5 mg) were dissolved in dimethyl sulfoxide (1 mL) and sterile water (9 mL) before mixing with 90 mL potato dextrose agar (PDA) to generate a final concentration of 50 μg/mL. Then, 4 mm diameter of the mycelia dishes were cut from a culture medium of pathogenic fungi, then inoculated in the middle of PDA and cultivated at 27 ± 1°C for 4–5 days. DMSO in sterile distilled water served as a negative control, while Kresoxim-methyl and Pyrimethanil acted as positive controls. For each treatment, three replicates were conducted. The radial growth of the fungal colonies was measured and the data were statistically analyzed. The inhibition rate *I* (%) of the test compounds against eight pathogenic fungi were calculated by the following formula, where *C* represents the diameter of fungi growth on untreated PDA, and *T* represents the diameter of fungi on treated PDA.

$$\begin{array}{l} I\left(\%\right)=\left[\left(C-T\right)/\left(C-0.4\right)\right]\times 100 \end{array}$$

#### Insecticidal Biological Assay

The insecticidal activities of all synthesized compounds **6a**–**6m** against *Spodoptera litura* (*S. litura*), *Mythimna separata* (*M. separata*)*, Pyrausta nubilalis* (*P. nubilalis*)*, Tetranychus urticae* (*T. urticae*)*, Rhopalosiphum maidis* (*R. maidis*), and *Nilaparvata lugens* (*N. lugens*) were performed according to the reported method (Wang B. L. et al., 2018; Wang et al., 2019). The target compounds **6a**–**6m** were dissolved in NP-10 (0.1 mg/L) solution to generate a final concentration of 200 μg/mL. Then, 15 maize leaves (approximately 5 cm in length) and 5 sweet potato leaves (diameter 3 cm) were dipped in the test compounds solutions for 10 s, dried and placed into a tumbler. After that, 30 larvae of second-instar *S. litura, M. separata, P. nubilalis, T. urticae, R. maidis*, and *N. lugens* were transferred to the petri dish. Chlorantraniliprole was used as a control. All bioassays were performed in the laboratory at 27 ± 1°C for 48 h. Three replicates were performed for each treatment. The percentage of mortalities for the target compounds were determined using Abbott's formula.

## Results and Discussion

In this study, using 2,3-dichloropyridine as the starting material, the title compounds **6a**–**6m** were synthesized in six steps, including hydrazidation, cyclization, bromination or chlorination, oxidation, hydrolyzation, and condensation. The target compounds structures were confirmed by ^1^H NMR, ^13^C NMR, MS, and elemental analysis. In the ^1^H NMR spectra of compound **6a**, a singlet at 2.45 ppm assigned to CH_3_ protons of pyrimidine-CH_3_, the doublet signal at 2.67 ppm indicated the presence of CH_3_ proton in CONH-CH_3_, meanwhile, a singlet at 8.78 and 7.32 ppm indicated the presence of pyrimidine and pyrazole ring. Two proton signals of two -CONH- in amide moiety was observed at 12.33 and 8.74 ppm. The structure of **6b** was also confirmed by its mass spectral data. In its mass spectrum, the molecular ion peak was noticed m/z at 450.1 ([M+H]^+^) corresponding to its molecular weight.

The *in vitro* antifungal activities at 50 μg/mL of the target compounds against eight plant fungi are listed in Table 1. Table 1 showed that, at 50 μg/mL, compounds **6a**–**6m** indicated certain antifungal activities against *S. sclerotiorum, P. infestans, T. cucumeris, G. zeae, F. oxysporum, C. mandshurica, B. dothidea*, and *Phompsis* sp. with the inhibition rates of 6.5–54.1%, 8.7–48.6%, 3.2–54.7%, 0–54.9%, 0–66.3%, 10.7–49.4%, 30.1–85.9%, and 36.2–81.2%, respectively. Among the title compounds, compound **6i** revealed good *in vitro* antifungal activities against *P. infestans* and *B. dothidea*, with the inhibition rates of 48.6% and 85.9%, respectively, which were equally to those of Kresoxim-methyl or Pyrimethanil. Meanwhile, compound **6j** revealed good *in vitro* antifungal activities against *S. sclerotiorum, G. zeae, C. mandshurica*, and *B. dothidea*, with the inhibition rates of 54.1, 54.9, 49.4, and 85.9%, respectively, which were similar with those of Kresoxim-methyl or Pyrimethanil.

**Table 1:** The *in vitro* antifungal activity of the title compounds 6a–6m at 50 μg/mL.

**Compds**.	**Inhibition rate (%)**							
	***S. sclerotiorum***	***P. infestans***	***T. cucumeris***	***G. zeae***	***F. oxysporum***	***C. mandshurica***	***B. dothidea***	***Phompsis sp*.**
**6a**	27.5 ± 1.1	20.7 ± 1.0	6.8 ± 1.9	0	16.9 ± 0.9	10.7 ± 1.1	31.6 ± 2.1	36.2 ± 1.4
**6b**	37.8 ± 1.6	29.6 ± 1.3	19.7 ± 1.4	8.7 ± 1.1	36.0 ± 1.3	40.2 ± 1.7	36.5 ± 1.6	42.1 ± 1.0
**6c**	35.0 ± 1.4	16.8 ± 1.3	14.2 ± 0.9	34.2 ± 1.3	12.1 ± 0.9	22.2 ± 1.0	45.9 ± 3.3	53.5 ± 1.2
**6d**	34.0 ± 1.3	8.7 ± 0.8	8.1 ± 1.2	0	2.6 ± 1.1	11.7 ± 0.9	50.8 ± 3.1	57.5 ± 2.1
**6e**	6.5 ± 0.9	10.0 ± 0.9	3.2 ± 1.0	9.9 ± 0.9	0	12.8 ± 3.0	30.1 ± 1.9	40.3 ± 2.7
**6f**	11.8 ± 3.8	15.1 ± 1.2	9.4 ± 1.2	7.8 ± 2.2	16.2 ± 1.7	21.2 ± 0.9	37.5 ± 3.0	46.2 ± 1.4
**6g**	41.5 ± 1.0	32.9 ± 1.0	35.2 ± 1.3	15.1 ± 1.5	32.1 ± 0.9	14.8 ± 0.9	38.4 ± 1.2	41.8 ± 3.3
**6h**	37.4 ± 1.2	27.0 ± 0.9	37.5 ± 1.0	21.6 ± 1.5	25.3 ± 1.9	20.7 ± 1.0	44.9 ± 3.0	46.3 ± 2.2
**6i**	47.9 ± 1.6	48.6 ± 1.4	49.2 ± 1.8	48.8 ± 1.6	66.3 ± 1.0	41.5 ± 1.4	85.9 ± 1.1	81.2 ± 2.4
**6j**	54.1 ± 1.1	42.5 ± 1.2	54.7 ± 2.3	54.9 ± 1.5	53.8 ± 1.3	49.4 ± 1.2	83.6 ± 2.1	78.8 ± 1.2
**6k**	39.9 ± 3.1	30.1 ± 0.9	47.9 ± 1.2	41.2 ± 1.1	45.6 ± 1.1	47.7 ± 1.3	55.6 ± 2.8	57.0 ± 1.9
**6l**	30.6 ± 2.3	24.1 ± 1.7	40.3 ± 2.6	33.7 ± 2.8	38.5 ± 2.3	40.2 ± 1.8	59.5 ± 3.0	62.2 ± 3.5
**6m**	24.8 ± 1.2	16.3 ± 1.4	34.6 ± 0.9	24.9 ± 1.7	32.2 ± 1.6	32.5 ± 1.6	46.2 ± 2.7	51.4 ± 2.0
Kresoxim-methyl	52.3 ± 1.1	49.8 ± 1.0	64.1 ± 1.2	56.9 ± 1.0	70.6 ± 1.2	51.1 ± 1.3	/	/
Pyrimethanil	/	/	/	/	/	/	84.4 ± 2.1	85.1 ± 1.4

Based on the preliminary antifungal bioassays, the EC_50_ values of compounds **6i** and **6j** were also tested and presented in Table 2. Table 2 showed that compounds **6i** and **6j** showed good activities against *B. dothidea*, with EC_50_ values of 56.4 and 65.3 μg/mL, respectively, which were similar to that of Pyrimethanil (57.6 μg/mL).

**Table 2:** The EC_50_ values of compounds 6i and 6j against *B. dothidea*.

**Compounds**	**Toxic regression equation**	***r***	**EC_**50**_ (μg/mL)**
**6i**	y = 1.21 × +3.56	0.99	56.4 ± 1.2
**6j**	y = 0.85x + 4.15	0.99	65.3 ± 1.1
Pyrimethanil	y = 1.01x + 6.25	0.99	57.6 ± 1.8

The *in vitro* insecticidal properties of title compounds against *S. litura, M. separata, P. nubilalis, T. urticae, R. maidis*, and *N. lugens* were evaluated and the insecticidal bioassay results are listed in Table 3. Table 3 showed that the target compounds **6a**–**6m** indicated certain insecticidal activities against *S. litura, M. separata, P. nubilalis, T. urticae, R. maidis*, and *N. lugens* at 200 μg/mL, with the mortality rates of 10.0–70.2%, 12.0–75.4%, 0–61.3%, 43.0–80.9%, 20.0–88.9%, and 11.0–78.6%, respectively, which were lower than those of Chlorantraniliprole. Especially, compound **6f** revealed good insecticidal activities against *S. litura, M. separata, P. nubilalis, T. urticae*, and *N. lugens* with mortality rates of 70.2, 75.4, 61.3, 80.9, and 78.6%, respectively. Meanwhile, compound **6c** had a good mortality rate of 88.9% against *R. maidis*.

**Table 3:** The insecticidal activities of the title compounds 6a–6m at 200 μg/mL.

**Compds**.	**Mortality rate (%)**					
	***S. litura***	***M. separata***	***P. nubilalis***	***T. urticae***	***R. maidis***	***N. lugens***
**6a**	21.8 ± 1.2	12.0 ± 2.5	25.6 ± 2.0	49.8 ± 0.9	20.0 ± 1.8	64.1 ± 2.6
**6b**	10.0 ± 1.0	48.5 ± 3.2	31.6 ± 1.5	59.6 ± 2.2	30.0 ± 2.2	74.6 ± 3.0
**6c**	11.8 ± 1.5	56.8 ± 2.8	28.1 ± 1.2	43.0 ± 2.6	88.9 ± 2.0	67.3 ± 2.8
**6d**	18.6 ± 2.0	16.7 ± 2.2	0	57.2 ± 2.5	22.4 ± 1.1	63.5 ± 2.5
**6e**	43.3 ± 2.5	66.3 ± 2.0	42.7 ± 2.3	51.6 ± 2.0	26.7 ± 1.5	50.3 ± 1.5
**6f**	70.2 ± 2.8	75.4 ± 2.8	61.3 ± 4.5	80.9 ± 4.5	65.5 ± 3.0	78.6 ± 3.3
**6g**	45.2 ± 2.0	19.6 ± 1.0	38.3 ± 1.1	57.2 ± 3.5	40.9 ± 2.0	41.6 ± 2.5
**6h**	37.5 ± 2.2	24.8 ± 1.5	46.9 ± 3.2	61.4 ± 2.8	36.5 ± 1.5	50.5 ± 3.1
**6i**	30.0 ± 1.8	26.6 ± 2.0	10.0 ± 2.2	52.7 ± 2.5	37.8 ± 1.2	28.4 ± 1.8
**6j**	35.3 ± 1.5	31.2 ± 2.8	26.1 ± 1.8	61.7 ± 3.0	43.2 ± 1.8	24.1 ± 1.5
**6k**	40.2 ± 1.4	32.0 ± 2.1	18.4 ± 1.5	72.6 ± 2.6	40.2 ± 1.5	32.5 ± 2.8
**6l**	32.4 ± 1.0	27.5 ± 1.5	23.5 ± 1.5	64.5 ± 2.2	33.8 ± 1.0	23.8 ± 1.9
**6m**	26.1 ± 0.8	23.4 ± 1.2	28.2 ± 1.0	57.8 ± 2.0	26.6 ± 0.8	11.0 ± 2.5
Chlorantraniliprole	100.0	100.0	100.0	100.0	100.0	100.0

## Conclusions

In summary, thirteen novel pyridylpyrazolamide derivatives containing pyrimidine motifs were synthesized, and their structures confirmed by ^1^H NMR, ^13^C NMR, MS and elemental analysis. Bioassay results showed that some of title compounds revealed good antifungal and insecticidal properties. The results provided strategy for leading the synthesis of novel pyridylpyrazolamide derivatives containing pyrimidine motifs.

## Data Availability Statement

The original contributions presented in the study are included in the article/Supplementary Material, further inquiries can be directed to the corresponding author/s.

## Author Contributions

WW and QF contributed to the synthesis, purification, characterization of all compounds, and prepared the original manuscript. YZ and YG performed the activity research. MC and HC perfected the language and assisted with the structure elucidation and manuscript revision. GO and MY designed and supervised the research and revised the manuscript. All authors discussed, edited, and approved the final version.

## Conflict of Interest

The authors declare that the research was conducted in the absence of any commercial or financial relationships that could be construed as a potential conflict of interest. The handling editor declared a shared affiliation, though no other collaboration, with the authors YZ, MY, and GO.
