# Supplementary material for: Synthesis and Bioactivities Study of Novel Pyridylpyrazol Amide Derivatives Containing Pyrimidine Motifs
Source: Front Chem. 2020 Jul 31;8:522. doi: 10.3389/fchem.2020.00522 (PMC7411148; doi:10.3389/fchem.2020.00522)
Supplement: Supplementary file 1 [file Data_Sheet_1.docx]

**Synthesis and Bioactivities Study of Novel Pyridylpyrazol Amide Derivatives Containing a Pyrimidine Moiety**

***Wenneng Wu^1,2^, Meihang Chen^3^, Qiang Fei^1^, Yonghui Ge^1^, Yingying Zhu^4^, Haijiang Chen^1^, Maofa Yang^2^*, Guiping Ouyang^2^****

*^1^Food and Pharmaceutical Engineering Institute, Guiyang University, Guiyang, China,* *^2^Entomology Institute, Center for Research and Development of Fine Chemicals, School of Pharmaceutical Sciences, Guizhou University, Guiyang, China,* *^3^Material and Chemistry Engineering Institute, Tongren College, Tongren , China,* *^4^School of Chemical Engineering, Guizhou Institute of Technology, Guiyang, China*

**Supplementary data**

**^1^H NMR spectral data for synthesized compounds 1-5**

3-Chloro-2-hydrazinylpyridine (**1**): white crystal, yield 90.0%; m.p. 161-162 ^o^C; ^1^H NMR (DMSO-*d*_6_, 500 MHz) *δ*: 8.05 (d, 1H, *J* = 3.5 Hz, Pyridine-H), 7.57 (d, 1H, *J* = 1.2 Hz, Pyridine-H), 6.62 (t, 1H, *J* = 3.7 Hz, Pyridine-H), 4.22 (s, 1H, NH), 2.51 (s, 1H, NH_2_).

Ethyl 2-(3-chloropyridin-2-yl)-5-oxopyrazolidine-3-carboxylate (**2**): yellow crystal, yield 52.0%; m.p. 132-134 ^o^C; ^1^H NMR (DMSO-*d*_6_, 500 MHz) *δ*: 10.22 (s, 1H, OH), 8.31 (d, 1H, *J* = 5.2 Hz, Pyridine-H), 7.80 (d, 1H, *J* = 1.2 Hz, Pyridine-H), 7.24 (t, 1H, *J* = 3.85 Hz, Pyridine-H), 4.87 (d, 1H, Parazole-H), 4.23 (q, 2H, *J* = 7.2 Hz), 1.26 (t, 3H, *J* = 7.2 Hz).

Ethyl 3-chloro-1-(3-chloropyridin-2-yl)-4,5-dihydro-1H-pyrazole-5-carboxylate (**3a**): amber oily; yield 85%; ^1^H NMR (DMSO-*d*_6_, 500 MHz) *δ*: 8.59 (d, 1H, *J* = 1.2 Hz, Pyridine-H), 8.26 (d, 1H, *J* = 1.2 Hz, Pyridine-H ), 7.73 (m, 1H, Pyridine-H), 5.40 ( m, 1H), 4.23 (q, 2H*, J* = 7.2 Hz), 3.49-3.53 (m, 2H), 1.26 ( t, 3H *, J* = 7.2 Hz);

Ethyl 3-bromo-1-(3-chloropyridin-2-yl)-4,5-dihydro-1H-pyrazole-5-carboxylate(**3b**): amber oily; yield 82%; ^1^H NMR ( DMSO-*d*_6_, 500 MHz) *δ*: 8.57 (d, 1H, *J* = 1.2 Hz, Pyridine-H), 8.25 ( d, 1H, *J* = 1.2 Hz, Pyridine-H ), 7.68 ( m, 1H, Pyridine-H), 5.34 ( dd, 1H), 4.23 (q, 2H, *J* = 7.2 Hz), 3.47-3.48 (m, 2H), 1.26 (t, 3H, *J* =7.2 Hz).

Ethyl 3-chloro-1-(3-chloropyridin-2-yl)-1H-pyrazole-5-carboxylate(**4a**): yellow crystal; yield 70%; m.p. 144-146 ^o^C ; 1H NMR (DMSO-*d*_6_, 500 MHz) *δ*: 8.57 (d, 1H, *J* = 1.2 Hz, Pyridine-H), 8.25 (d, 1H, *J* = 1.2 Hz, Pyridine-H), 7.68 (dd, 1H, *J_1_* = 6.5 Hz, *J_2_* = 6.8 Hz, Pyridine-H), 7.21 (s, 1H, Parazole-H), 4.23 (q, 2H, *J* =7.2 Hz), 1.26 (t, 3H, *J* = 7.2 Hz);

Ethyl 3-bromo-1-(3-chloropyridin-2-yl)-1H-pyrazole-5-carboxylate(4a): yellow crystal; yield 80%; m.p. 117-118 ^o^C; ^1^H NMR (DMSO-*d*_6_, 500 MHz) *δ*: 8.60 (d, 1H, *J* = 1.2 Hz, Pyridine-H), 8.30 (d, 1H, *J* = 1.2 Hz, Pyridine-H), 7.69 (dd, 1H, *J_1_* = 6.5 Hz, *J_2_* = 6.8 Hz, Pyridine-H), 7.24 (s, 1H, Parazole-H), 4.23 (q, 2H, *J* = 7.2 Hz), 1.26 (t, 3H, *J* = 7.2 Hz).

3-Chloro-1-(3-chloropyridin-2-yl)-1H-pyrazole-5-carboxylic acid(**5a**): yellow crystal; yield 80 %; m.p. 207-208 ^o^C; ^1^H NMR (DMSO-*d*_6_, 500 MHz) *δ*: 13.99 (s, 1H, COOH), 8.57 (d, 1H, *J* = 1.2 Hz, Pyridine-H), 8.25 (d, 1H, *J* = 1.2 Hz, Pyridine-H), 7.68 (dd, 1H, *J_1_* = 6.5 Hz, *J_2_* = 6.8 Hz, Pyridine-H), 7.21 (s, 1H, Parazole-H).

3-Bromo-1-(3-chloropyridin-2-yl)-1H-pyrazole-5-carboxylic acid(5a): yellow crystal; yield 70 %; m.p. 200-201 ^o^C; ^1^H NMR (DMSO-*d*_6_, 500 MHz) *δ*: 13.87 (s, 1H, COOH), 8.60 (d, 1H, *J* = 1.2 Hz, Pyridine-H), 8.30 (d, 1H, *J* = 1.2 Hz, Pyridine-H), 7.69 (dd, 1H, *J_1_* = 6.5 Hz, *J_2_* = 6.8 Hz, Pyridine-H), 7.24 (s, 1H, Parazole-H).
